# Comparative expression profiling of testis-enriched genes regulated during the development of spermatogonial cells

**DOI:** 10.1371/journal.pone.0175787

**Published:** 2017-04-17

**Authors:** Jinsoo Ahn, Yoo-Jin Park, Paula Chen, Tae Jin Lee, Young-Jun Jeon, Carlo M. Croce, Yeunsu Suh, Seongsoo Hwang, Woo-Sung Kwon, Myung-Geol Pang, Cheorl-Ho Kim, Sang Suk Lee, Kichoon Lee

**Affiliations:** 1 Department of Animal Sciences, The Ohio State University, Columbus, Ohio, United States of America; 2 Center for Systems Biology, Program in Membrane Biology/Nephrology Division, Massachusetts General Hospital, Boston, MA and Harvard Medical School, Boston, Massachusetts, United States of America; 3 Department of Cancer Biology and Genetics, The Ohio State University, Columbus, Ohio, United States of America; 4 Stanford Cancer Institute, Stanford University, Stanford, California, United States of America; 5 Animal Biotechnology Division, National Institute of Animal Science, RDA, Wanju-gun, Jeonbuk, Republic of Korea; 6 Department of Animal Biotechnology, Kyungpook National University, Sangju, Republic of Korea; 7 Department of Animal Science and Technology, Chung-Ang University, Anseong, Gyeonggi-do, Republic of Korea; 8 Department of Biological Sciences, SungKyunKwan University, Chunchun-Dong, Jangan-Gu, Suwon City, Kyunggi-Do, Republic of Korea; 9 Department of Animal Science and Technology, Sunchon National University, Suncheon, Republic of Korea; Shanghai Ocean University, CHINA

## Abstract

The testis has been identified as the organ in which a large number of tissue-enriched genes are present. However, a large portion of transcripts related to each stage or cell type in the testis still remains unknown. In this study, databases combined with confirmatory measurements were used to investigate testis-enriched genes, localization in the testis, developmental regulation, gene expression profiles of testicular disease, and signaling pathways. Our comparative analysis of GEO DataSets showed that 24 genes are predominantly expressed in testis. Cellular locations of 15 testis-enriched proteins in human testis have been identified and most of them were located in spermatocytes and round spermatids. Real-time PCR revealed that expressions of these 15 genes are significantly increased during testis development. Also, an analysis of GEO DataSets indicated that expressions of these 15 genes were significantly decreased in teratozoospermic patients and polyubiquitin knockout mice, suggesting their involvement in normal testis development. Pathway analysis revealed that most of those 15 genes are implicated in various sperm-related cell processes and disease conditions. This approach provides effective strategies for discovering novel testis-enriched genes and their expression patterns, paving the way for future characterization of their functions regarding infertility and providing new biomarkers for specific stages of spematogenesis.

## Introduction

The testis has been identified by RNA sequencing as the organ in which the largest number of tissue-enriched genes is expressed among various organs. It has been estimated that expressions of more than 1000 genes are enriched in the testis [1]; whereas, on average, there are approximately 200 signature genes in each tissue [2]. Tissue-enriched or tissue-specific genes are essential for the growth and development of specific cells and organs [3]. Thus, characteristic processes that occurred in germinal cells in the testis, including meiosis, genetic recombination, spermatogenesis, and spermiogenesis may largely be attributed to a number of differential gene expressions. Spermatogenesis is a complex process that is orchestrated by expression of multiple genes at various stages containing particular cell types, such as spermatogonial stem cells, spermatogonia, spermatocytes, and spermatids [4]. In addition to germinal cells, the somatic Sertoli cells play a role in testis formation and provide an essential environment for spermatogenesis [5], and Leydig cells produce androgen, which plays a key role in the regulation of spermatogenesis and undergo changes in gene expression [6, 7]. However, a large portion of transcripts and proteins related to each stage or cell type as well as their functions still remains unknown.

Investigation of gene expression and function during spermatogenesis has been hampered by a lack of immortalized cell lines for each stage [8]. Alternatively, testis transcriptome microarray analysis based on Gene Expression Omnibus (GEO) repository (www.ncbi.nlm.nih.gov/geo) followed by protein profiling using immunohistochemical data from the Human Protein Atlas portal (www.proteinatlas.org) is a useful tool for discovering highly expressed genes in each stage of spermatogenesis in the testis. Furthermore, gene expression profiles under various developmental, disease, and knockout conditions produced in GEO microarray datasets offer a platform for functional genomic studies of spermatogenesis stage-specific gene expression.

Using these sources combined with confirmatory gene expression measurements and pathway analysis, in this study, protein localization and signaling pathways of 15 testis-enriched genes were analyzed. The objectives of this study were to identify novel testis-enriched genes using gene expression profiles and analyze protein localization, developmental regulation and biological implications of testis-enriched genes in humans and mice. The current approach provides an effective strategy for discovering novel testis-enriched genes and their unique stage-specific expression, paving the way for future studies of normal development of the testis and associated diseases.

## Materials and methods

### Microarray data mining

The microarray-based, high-throughput gene expression data were obtained from the GDS DataSet (GDS) of the GEO repository in the National Center for Biotechnology Information (NCBI) archives (www.ncbi.nlm.nih.gov/geo). For analyzing tissue distribution pattern of gene expression in 12 male mouse tissues and 10 man tissues, GDS3142 for mice and GDS596 for humans were downloaded and sorted (Tables 1 and 2) as described in our previous reports [9, 10]. Also, gene expression patterns in mouse sperm cells (GDS2390), developing mouse testis (GDS605, GDS606 and GDS607), semen samples collected from 14 teratozoospermic individuals aged 21–57 (GDS2697), and polyubiquitin knockout mice (GDS3906) were examined.

**Table 1:** Mouse testis-enriched genes based on GDS3142.

Gene	Fold	Testis	Ovary	Muscle	Liver	Brain	Lung	Kidney	Adipose	Thymus	Heart	Spleen	SI	*P* value	Testis enrichment	Location in mouse testis
*Tnp1*	**276**	**21266**	71	72	91	78	81	81	66	76	77	74	81	< .0001	[43]	‒
*Ldhc*	**242**	**16560**	73	70	61	64	65	65	59	72	87	59	78	< .0001	[13]	[14, 15]
*Prm1*	**214**	**12302**	63	56	54	54	54	55	55	63	62	56	60	< .0001	[44]	[45]
*Prm2*	**176**	**20955**	99	125	109	99	107	104	100	134	176	100	156	< .0001	[46]	[46]
*Akap3*	**139**	**11583**	75	80	85	74	79	80	70	92	112	68	100	< .0001	[47]	[47]
*Smcp*	**125**	**10373**	70	73	74	66	69	163	71	80	93	74	79	< .0001	**Fig 1**	[48–50]
*Odf1*	**117**	**11076**	82	90	100	80	90	99	94	106	110	82	109	< .0001	**Fig 1**	‒
*Crisp2*	**110**	**7771**	65	66	70	69	68	63	73	67	81	83	71	< .0001	**Fig 1**	‒
*Tcfl5*	**98**	**7086**	73	69	70	88	66	71	68	70	78	67	75	< .0001	[51]	[51]
*Odf2*	**76**	**7462**	108	96	75	118	99	93	86	123	88	94	104	< .0001	[52]	[52]
*Phf7*	**69**	**13455**	174	318	196	118	141	219	170	251	166	216	169	< .0001	**Fig 1**	[17]
*Tcp11*	**47**	**7904**	146	180	177	132	159	157	156	196	173	157	207	< .0001	[53]	[53]
*Actl7b*	**40**	**6562**	120	144	167	142	166	135	135	161	356	128	173	< .0001	[54]	[54]
*Ybx2*	**37**	**5221**	137	147	110	104	109	146	135	160	235	101	157	< .0001	[55]	‒
*Gapdhs*	**36**	**5894**	121	182	145	103	107	133	142	230	290	110	240	< .0001	**Fig 1**	‒
*Spink2*	**35**	**4130**	104	118	120	99	124	102	133	126	149	97	136	< .0001	[20]	[20]
*Zpbp*	**26**	**2178**	81	77	79	79	79	82	84	94	106	80	91	< .0001	[26]	[56]
*Spata6*	**23**	**3652**	168	112	89	83	205	208	171	260	154	156	113	< .0001	[57, 58]	[57, 58]
*Actl7a*	**23**	**2628**	101	119	112	92	97	94	105	143	183	104	128	< .0001	[54, 59]	[54]
*Ddx4*	**21**	**1350**	85	56	57	75	57	59	61	62	61	62	58	< .0001	**Fig 1**	‒
*Efhc1*	**19**	**2126**	234	80	84	77	196	74	94	105	104	73	90	< .0001	[23]	[23]
*Nek2*	**18**	**3008**	214	68	75	72	124	88	82	627	74	169	285	< .0001	[60, 61]	[60, 61]
*Stag3*	**16**	**1324**	81	72	85	71	73	75	77	112	84	86	97	< .0001	[62, 63]	‒
*Zmynd10*	**15**	**2133**	145	123	123	111	149	291	102	100	148	109	122	< .0001	**Fig 1**	‒

**Table 2:** Human testis-enriched genes based on GDS596.

Gene	Fold	Testis	Ovary	Muscle	Liver	Brain	Lung	Kidney	Adipocyte	Thymus	Heart	*P* value	Testis enrichment	Location in human testis	Location in mouse Testis (Table 1)
*PRM2*	**861**	**12387**	6	55	13	12	8	12	7	4	13	< .0001	**Fig 1**	[64]	[46]
*PRM1*	**800**	**37144**	42	135	24	11	63	47	15	11	68	< .0001	[65]	[64]	[45]
*TNP1*	**186**	**17960**	27	284	66	22	47	135	94	21	174	< .0001	**Fig 1**	[66]	‒
*ODF2*	**181**	**4179**	6	67	27	13	12	18	16	11	37	< .0001	[67]	[67]	[52]
*SPATA6*	**107**	**1742**	8	44	15	8	7	22	13	9	20	< .0001	**Fig 1**	**Fig 2B**	[57, 58]
*PHF7*	**77**	**7891**	82	417	47	77	47	62	53	46	86	< .0001	[17]	**Fig 2A**	[17]
*CRISP2*	**68**	**4526**	53	396	43	25	8	10	47	4	14	< .0001	[68]	[69]	‒
*SPINK2*	**63**	**7706**	52	257	134	86	106	67	63	125	215	< .0001	[70]	**Fig 2A**	[20]
*NEK2*	**47**	**1427**	28	71	13	29	7	16	7	75	28	< .0001	**Fig 1**	[71]	[60, 61]
*LDHC*	**46**	**3625**	40	254	70	22	38	19	134	25	102	< .0001	**Fig 1**	**Fig 2A**	[14, 15]
*SMCP*	**39**	**2082**	25	172	53	29	15	64	31	18	76	< .0001	[72]	[72]	[48–50]
*YBX2*	**37**	**8120**	67	719	224	258	70	337	108	46	160	< .0001	**Fig 1**	**Fig 2C**	‒
*ZPBP*	**35**	**3012**	87	128	50	64	73	74	24	31	236	< .0001	[26]	**Fig 2B**	[56]
*ACTL7A*	**35**	**1938**	17	245	38	27	21	59	28	16	55	< .0001	[73]	**Fig 2B**	[54]
*TCP11*	**33**	**7549**	37	632	227	160	122	293	196	78	340	< .0001	[74]	**Fig 2A**	[53]
*ZMYND10*	**32**	**1505**	10	165	42	18	29	66	22	8	60	< .0001	[75]	**Fig 2C**	‒
*ACTL7B*	**30**	**1876**	41	184	79	73	17	38	62	37	27	< .0001	[73]	**Fig 2B**	[54]
*ODF1*	**25**	**5549**	183	493	223	130	80	300	145	72	361	< .0001	[76]	**Fig 2D**	‒
*AKAP3*	**24**	**1166**	23	120	59	39	33	61	18	16	69	< .0001	[77–79]	[77]	[47]
*GAPDHS*	**21**	**3357**	29	250	216	192	86	96	120	73	401	< .0001	[80]	**Fig 2D**	‒
*DDX4*	**19**	**447**	7	115	6	6	3	33	5	4	27	< .0001	[81]	[81]	‒
*TCFL5*	**14**	**2611**	11	259	79	562	109	251	216	182	57	< .0001	[82]	**Fig 2A**	[51]
*STAG3*	**13**	**1885**	44	216	140	130	92	217	57	224	141	< .0001	[62]	**Fig 2C**	‒
*EFHC1*	**13**	**407**	18	98	53	16	10	32	13	29	14	< .0001	**Fig 1**	**Fig 2A**	[23]

### Animal use and sample preparation

All animal care and procedures were approved by the Institutional Animal Care and Use Committee (IACUC) at The Ohio State University. Mice were raised under ad libitum feeding conditions in a mice housing facility at The Ohio State University. Mice were euthanized by carbon dioxide inhalation followed by cervical dislocation. For isolation of total RNAs, testis, muscle, liver, brain, lung, kidney, adipose tissue, thymus, spleen, and small intestine were collected from 3-month-old FVB mice (n = 3) using Trizol reagent (Invitrogen, Carlsbad, CA, USA) [11]. Total RNAs from the adult human kidney, liver, lung, heart, muscle, testis, thymus, and brain were purchased from Agilent Technologies (Santa Clara, CA, USA) and adult human RNA from adipose tissue was purchased from Clontech Laboratories (Mountain View, CA, USA). For RNA isolation from mouse testis at 10 days postpartum (dpp), 21 dpp, and 91 dpp (three months postpartum), C57BL/6 mice (n = 4) were euthanized and both testes were harvested.

### Reverse transcription PCR (RT-PCR)

To measure the quantity of RNA, a Nanodrop spectrophotometer (Thermo Scientific, Wilmington, DE) was used. The RNA samples were stored at -80°C until use. Approximately 1 μg of RNA was reverse-transcribed in a 20 μL total reaction to cDNA using Moloney murine leukemia virus (M-MLV) reverse transcriptase (Invitrogen). The thermal cycle of the reverse transcription was 65°C for 5 min, 37°C for 52 min, and 70°C for 15 min. Exactly 1 μL of cDNA samples was used as a template for PCR in a 25 μL total reaction with AmpliTaq Gold DNA polymerase (Applied Biosystems, Carlsbad, CA). The conditions for this reaction were 95°C for 1 min 30 s, 33 cycles of 94°C for 30 s, 55°C for 1 min, 72°C for 1 min, with an additional extension step at 72°C for 10 min. PCR products were separated by using 1% agarose gel electrophoresis. Forward and reverse primers for both humans and mice listed in supporting information were designed on different exons for multi-exon genes to avoid genomic DNA contamination.

### Analysis of protein expression profiles from the Human Protein Atlas

Data visualizing immunohistochemically the expression patterns of selected proteins in human testes were obtained from the Human Protein Atlas portal (www.proteinatlas.org). A total of 15 testis-enriched proteins were analyzed for their localization in the human testis: ten of them have been published in terms of their localization in mouse testis, but not in human testis, and the rest of them have not been published regarding their localization in both human and mouse testis.

### Real-time PCR

Quantitative real-time PCR (qPCR) was performed on an ABI 7300 Real-Time PCR instrument (Applied BioSystems, Foster City, CA) by using AmpliTaq Gold polymerase (Applied BioSystems) with SYBR green detection dye. Cyclophilin (*CYC*) was used as a housekeeping gene. Reactions were performed in duplicate 25μL volumes and conditions for the qPCR were 95°C for 10 minutes followed by 40 cycles of 94°C for 15 seconds, 60°C for 40 seconds, 72°C for 30 seconds, and 82°C for 33 seconds. Relative quantification of gene expression was determined by using the 2^-ΔΔ^C_T_ method [12].

### Signaling pathway analysis

Signaling pathways of spermatocyte- or spermatid-enriched proteins were analyzed using Pathway Studio (v 11.2.5.9, Elsevier, Amsterdam, Netherland). A list of 15 testis-enriched proteins was entered into Pathway Studio. The resulting pathways were verified through the PubMed/Medline hyperlink embedded in each node.

### Statistical analysis

For comparison of gene expression in testis versus other tissues, one-way ANOVA followed by a Fisher’s protected least significant difference test was performed using SAS version 9.2 (SAS Institute Inc., Cary, NC). A Student’s *t* test was conducted to compare the difference between two means. Comparison of multiple means was conducted by one-way ANOVA followed by a Tukey’s post hoc test. The significance level was set at *p* < 0.05.

## Results

### Microarray analyses identified common testis-enriched genes for the mouse and human

**Fig 1:**
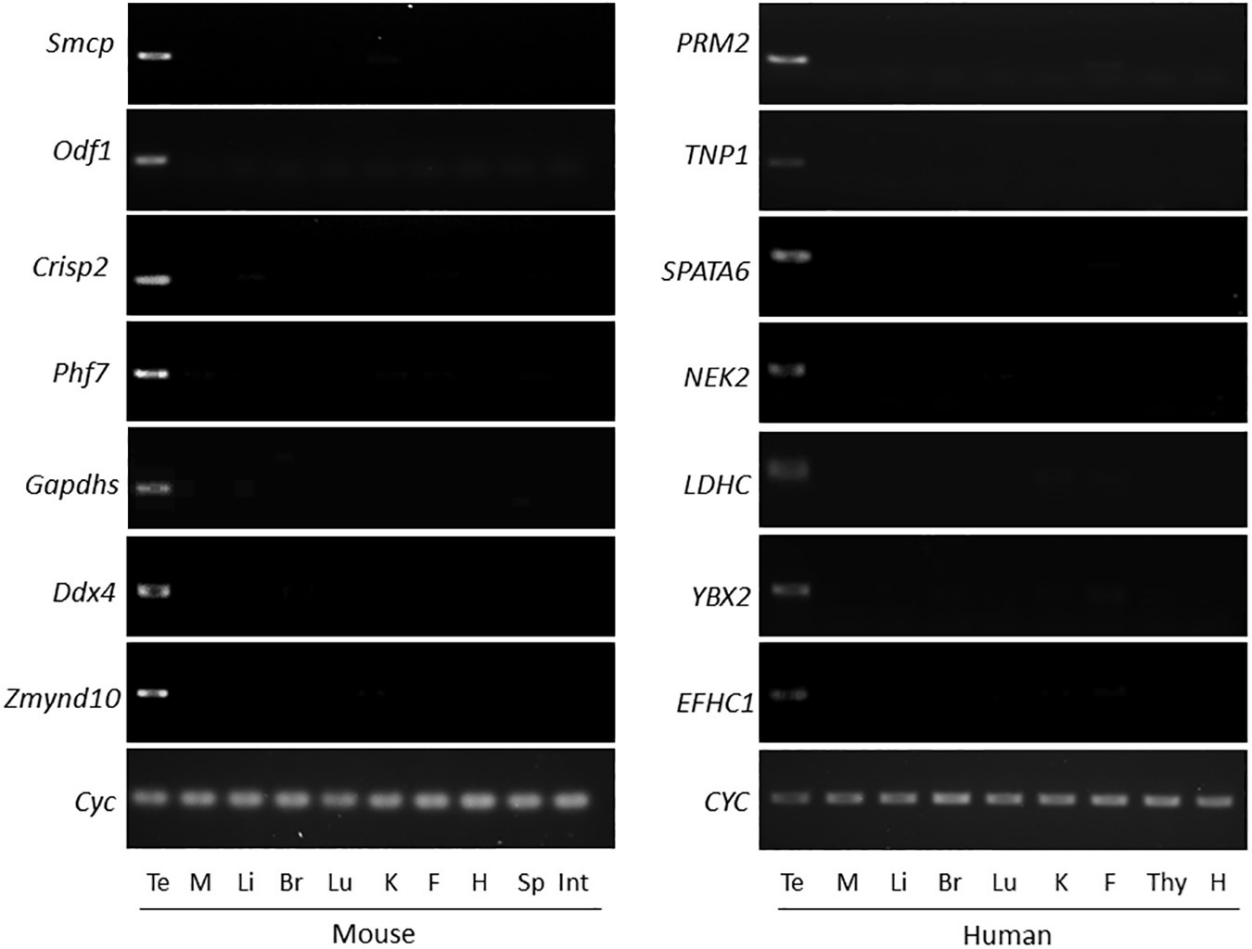
RT-PCR of mouse and human testis-enriched genes. Expression of mouse and human testis-enriched genes in various tissues are presented. Te: testis, M: muscle, Li: liver, Br: brain, Lu: lung, K: kidney, F: fat, H: heart, Sp: spleen, Int: intestine, Thy: thymus. Murine cyclophilin (*Cyc*) and human cyclophilin (*CYC*) genes were used as loading controls for an equal amount of cDNA.

**Fig 2:**
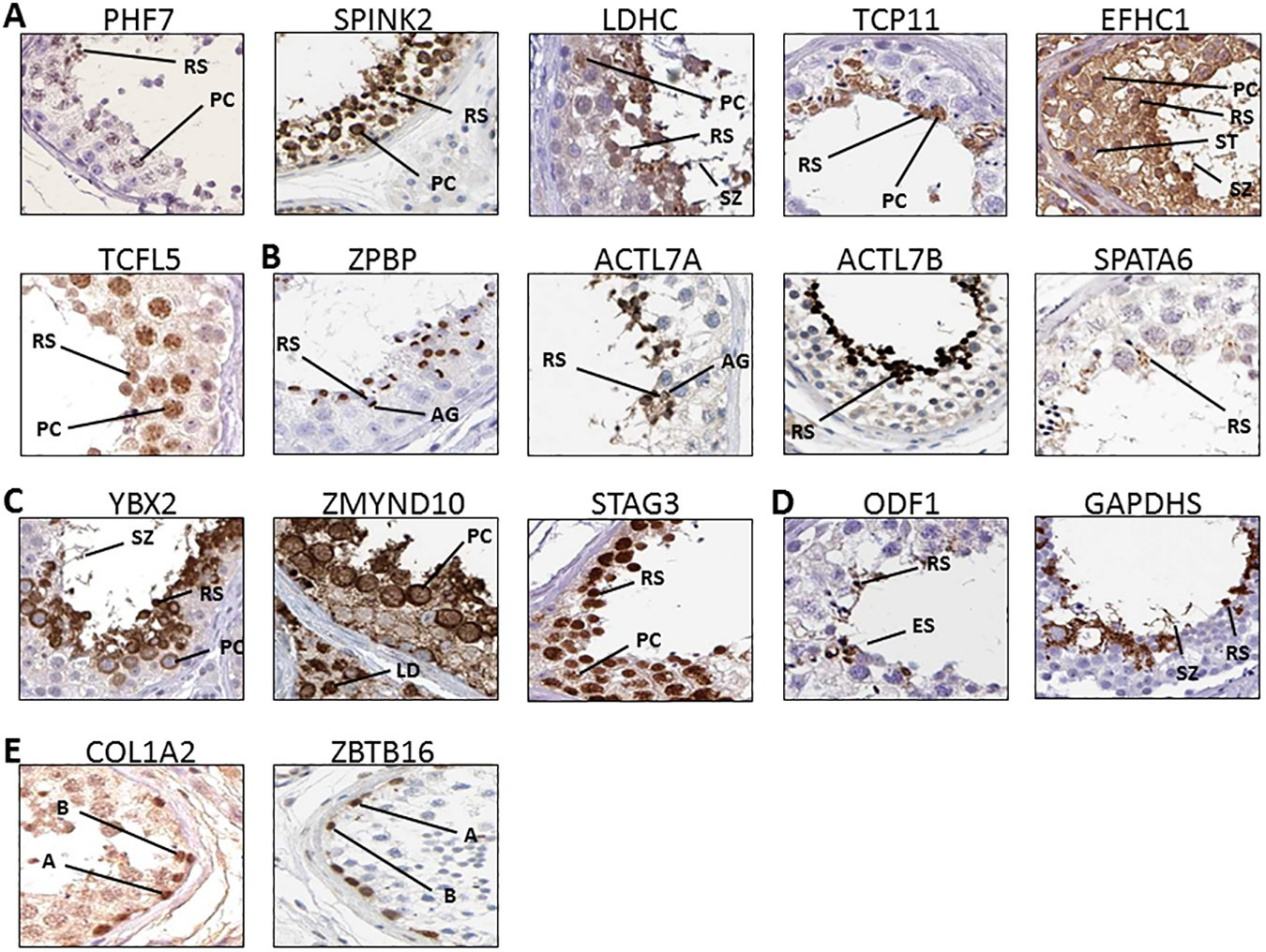
Immuno-localization of testis-specific genes in human testis based on figures obtained from the Human Protein Atlas (www.proteinatlas.org). Among genes that have been published regarding their localization in mouse testis, but not in human testis, proteins expressed in both pachytene spermatocytes and round spermatids (A) and in round spermatids (B) are described. Genes that have not been published regarding their localization in both human and mouse testis are displayed for their expression in both pachytene spermatocytes and round spermatids (C) and in round spermatids (D). In addition, proteins that are expressed in early spermatogenic cells (type A and B spermotogonia) are presented as non-testis-specific controls (E). A: type A spermatogonia, B: type B spermatogonia, PC: pachytene spermatocytes, RS: round spermatids, ES: elongating spermatids, AG: acrosomal granule, SZ: spermatozoa, ST: sertoli cells, LD: Leydig cells.

Comparative analysis of GEO DataSets (GDS3142 for mice and GDS596 for humans), a public microarray repository, revealed that expressions of 24 genes in both the mouse and human testis are more than 10-fold higher than an average expression value of other tissues (Tables 1 and 2). For example, murine *Tnp1* and human *TNP1* expressions are 276- and 186-fold greater in the mouse and human testis, respectively, than an average value of other tissues. In addition, these 24 genes are expressed at very low levels in the ovary, showing that they are male-specific genes. Our literature search revealed that some, but not all, genes were reported for testis enrichment and protein cellular location in testis. For instance, testis-specific expression of murine *Ldhc* gene and cellular protein location of LDHC were reported in the mouse testis [13–15], but not in human testis. In this study, testis enrichment of selected genes was confirmed by RT-PCR (Fig 1) and their localization profiles in humans were explored through the Human Protein Atlas (Fig 2).

### RT-PCR confirmed testis enrichment of selected genes

To validate the microarray data, RT-PCR was performed for murine *Smcp*, *Odf1*, *Crisp2*, *Phf7*, *Gapdhs*, *Ddx4* and *Zmynd10* and human *PRM2*, *TNP1*, *SPATA6*, *NEK2*, *LDHC*, *YBX2* and *EFHC1*, which have not been reported previously for expression in the testis. To prevent PCR saturation effects during amplification, the number of PCR cycles was reduced until the saturation no longer occurs. These genes showed testis-enriched expression patterns among various tissues (Fig 1), which is consistent with the GEO DataSets (Tables 1 and 2).

### Analysis of immunohistochemical data showed protein expression in specific stages of human testis

With the very latest version of the Human Protein Atlas, cellular location of several testis-enriched genes in humans was analyzed. When these locations were reported in mouse, they were grouped in Fig 2A and 2B; otherwise they were grouped in Fig 2C and 2D. As shown in Fig 2, PHF7, SPINK2, LDHC, TCP11, EFHC1, and TCFL5 proteins were located in earlier stage cells (Fig 2A) than cells expressing ZPBP, ACTL7A, ACTL7B, and SPATA6 (Fig 2B). PHF7, SPINK2, LDHC, TCP11, EFHC1, and TCFL5 were localized in pachytene spermatocytes (PC) and round spermatids (RS). In detail, SPINK2 was expressed strongly in the cytoplasm of pachytene spermatocytes, LDHC showed expression in the tail of spermatozoa (SZ), and EFHC1 was highly expressed in Sertoli cells (ST) (Fig 2A). ZPBP was uniquely detected in the developing acrosomal granules (AG) of round spermatids. ACTL7A was expressed in round spermatids and exclusively in the acrosome granules, with a lesser degree in spermatozoa tails. ACTL7B showed a stronger expression than SPATA6 in round spermatids (Fig 2B).

Other testis-enriched proteins shown in Fig 2C and 2D, such as YBX2, ZMYND10, STAG3, ODF1, and GAPDHS, have not been published regarding their localization in both human and mouse testis. YBX2 and ZMYND10 were strongly localized in the cytoplasm of pachytene spermatocytes (PC) and, to a lesser degree, the nucleus of pachytene spermatocytes in the case of ZMYND10. STAG3 was expressed in the nucleus of pachytene spermatocytes. These proteins were also present in round spermatids (RS) except for low expression of ZMYND10 in round spermatids. In addition, YBX2 was detected in the tail of spermatozoa (SZ), and ZMYND10 showed expression in Leydig cells (LD) (Fig 2C). ODF1 and GAPDHS were expressed in round spermatids (RS). Also, GAPDHS showed expression in the tail of spermatozoa (SZ). ODF1 was expressed in elongating spermatids (ES) with developing tails (Fig 2D).

In summary, most testis-enriched proteins selected in this study are expressed after the spermatogonia stage. Their gene expression profiles curated in GDS2390 also showed a significant increase of expression during the stages of pachytene spermatocytes and round spermatids (Table 3). On the other hand, two non-testis-enriched proteins, COL1A2 and ZBTB16, were mainly expressed in the early-stage cells such as type A and type B spermatogonia (Fig 2E) and similarly, their gene expression profiles showed significantly higher mRNA expression in spermatogonia stages (Table 3). Therefore, whether expression of testis-enriched genes is regulated during testis development was further analyzed.

**Table 3:** Differential gene expression in four types of spermatic cells according to GDS2390.

Gene	Type A spermatogonia	Type B spermatogonia	Pachytene spermatocytes	Round spermatids
**Testis-enriched genes**				
*Phf7*	62.3 ± 4.3^b^	385.0 ± 327.7^ab^	2877.8 ± 316.3^ab^	3073.8 ± 14.9^a^
*Spink2*	36.2 ± 4.7^b^	414.7 ± 392.7^ab^	2576.3 ± 181.8^a^	2527.2 ± 45.7^a^
*Ldhc*	34.5 ± 5.1^b^	2462.1 ± 2433.8^ab^	6269.1 ± 294.1^a^	6783.0 ± 205.1^a^
*Tcp11*	87.1 ± 4.6^c^	435.2 ± 356.3^bc^	2659.4 ± 144.6^b^	4347.4 ± 19.3^a^
*Efhc1*	66.1 ± 5.5^b^	288.3 ± 242.1^ab^	2012.3 ± 19.5^ab^	2476.6 ± 69.3^a^
*Tcfl5*	635.4 ± 68.0^c^	943.9 ± 247.0^c^	2967.8 ± 81.8^a^	2408.3 ± 77.9^b^
*Zpbp*	24.1 ± 1.4^bc^	204.4 ± 164.8^c^	1629.2 ± 224.3^ab^	3194.8 ± 49.0^a^
*Actl7a*	15.2 ± 4.0^b^	48.8 ± 36.0^b^	131.7 ± 22.6^b^	2097.9 ± 28.8^a^
*Actl7b*	58.8 ± 11.2^c^	253.1 ± 180.5^bc^	1120.6 ± 67.4^b^	3337.9 ± 223.4^a^
*Spata6*	47.2 ± 1.8^c^	133.3 ± 68.9^c^	784.1 ± 14.7^b^	2767.5 ± 143.3^a^
*Ybx2*	131.7 ± 39.9^b^	651.6 ± 508.2^ab^	3982.7 ± 107.3^a^	4034.0 ± 130.1^a^
*Zmynd10*	2.4 ± 0.6^c^	323.0 ± 321.6^bc^	2457.2 ± 121.3^a^	997.2 ± 44.6^b^
*Stag3*	725.9 ± 34.6^b^	984.0 ± 124.2^b^	2231.6 ± 124.7^a^	1762.4 ± 169.8^ab^
*Odf1*	3.7 ± 0.4^b^	62.0 ± 58.0^b^	275.9 ± 69.5^b^	3558.2 ± 185.0^a^
*Gapdhs*	71.3 ± 8.8^c^	122.1 ± 71.8^bc^	270.3 ± 0.3^b^	3604.4 ± 141.2^a^
**Non-testis-enriched genes**				
*Col1a2*	2743.6 ± 314.6^a^	3338.4 ± 173.2^a^	111.0 ± 18.6^b^	146.4 ± 1.0^b^
*Zbtb16*	304.7 ± 42.8^a^	178.9 ± 56.9^ab^	21.0 ± 1.2^b^	33.8 ± 13.0^b^

Means ± SEM are shown. Different superscript letters indicate significant differences between types of cells.

### Expression of testis-enriched genes showed an increasing pattern during normal testis development

**Fig 3:**
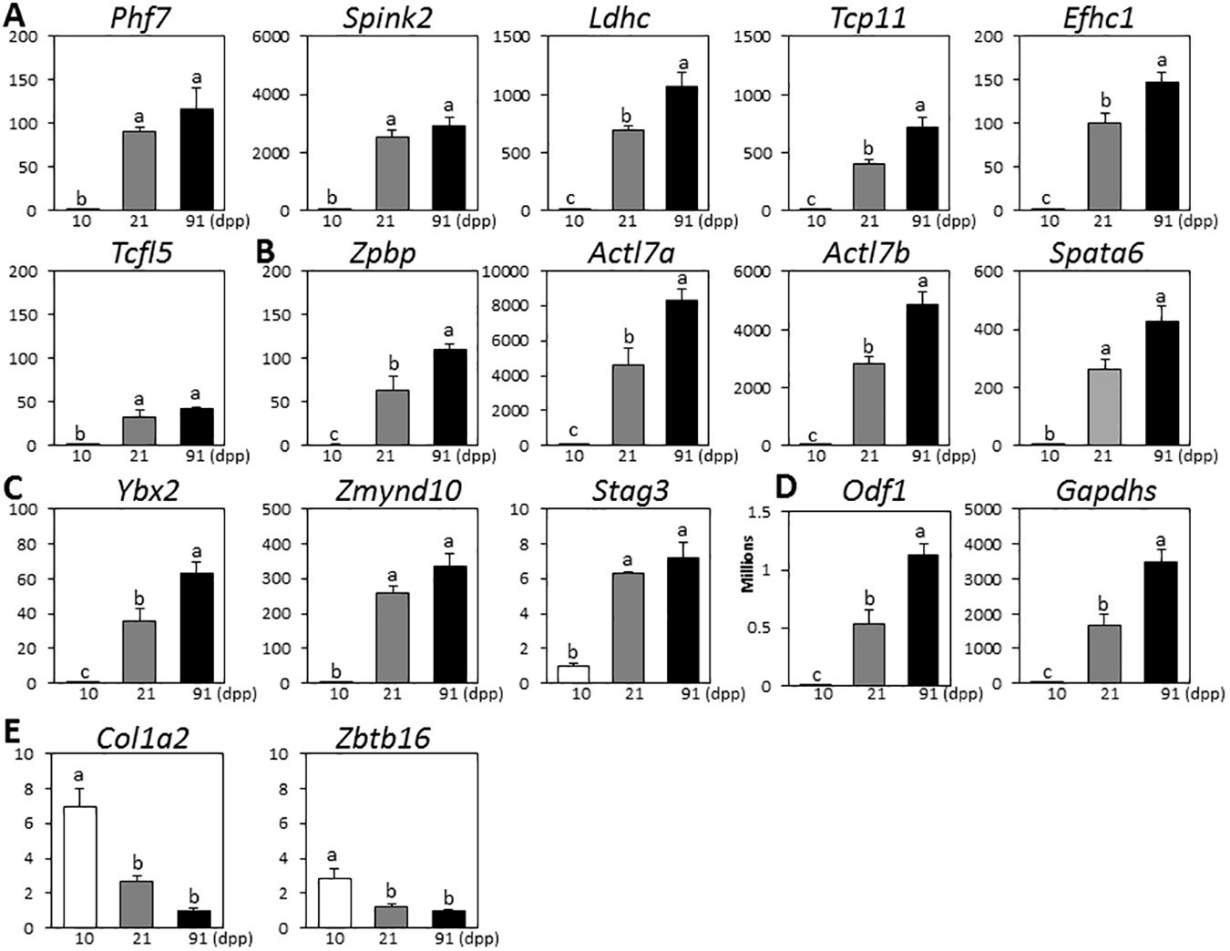
Real-time PCR analysis of developmental expression patterns of testis-specific genes in mouse testis. Quantitative real-time PCR (qPCR) results are presented for testis-related genes which are described in Fig 2 at 10, 21 and 91 days postpartum (dpp). According to categories of Fig 2, genes expressed in both pachytene spermatocytes and round spermatids (A) and in round spermatids (B) are presented for expression patterns. Among genes that have not been published for localization, genes expressed in both pachytene spermatocytes and round spermatids (C) and in round spermatids (D) are examined for expression patterns. In addition, non-testis-specific controls that are expressed in early spermatogenic cells (type A and B spermotogonia) are presented (E). The Y-axis represents relative expression value using cyclophilin (*Cyc*) as a housekeeping control. Each bar represents mean ± SEM. To compare means, one-way ANOVA was followed by Tukey’s post hoc test. Different letters above the bars indicate significant differences between developmental time points.

qPCR revealed that expression of selected testis-enriched genes is significantly increased during testis development. To investigate stage-specific expression patterns, 10 days postpartum (dpp) with mostly spermatogonia, 21 dpp when spermatocytes are the most abundant cell type, and 91 dpp representing an adult stage with spermatids were selected. Compared to 10 days postpartum (dpp), expression of these selected genes was significantly increased at 21 dpp after weaning and/or at 91 dpp (three months postpartum) when sexual maturation occurs (Fig 3A–3D). These data are consistent with the microarray database in GDS605, GDS606, and GDS607, which also shows an increasing pattern of these genes during the period of 0 through 35 dpp (Table 4). It suggests that expression of these testis-enriched genes is up-regulated during testis development and plays a role in later stages of spermatogenesis. In contrast, expression of both *Col1a2* and *Zbtb16* was significantly decreased at 21 dpp (Fig 3E), and this pattern was also shown in GDS605, GDS606, and GDS607 (Table 4), suggesting that these non-testis-enriched genes may be involved in early stages of spermatogenesis rather than the later stages.

**Table 4:** Differential gene expression during testis development based on GDS605, GDS606 and GDS607.

Gene	0–3 dpp	8–10 dpp	18–20 dpp	30–35 dpp
**Testis-enriched genes**				
*Phf7*	151.7 ± 22.5^b^	153.2 ± 7.2^b^	5045.2 ± 421.8^a^	6208.0 ± 450.3^a^
*Spink2*	122.5 ± 8.7^b^	99.1 ± 8.6^b^	1708.5 ± 221.6^a^	2506.2 ± 596.5^a^
*Ldhc*	48.4 ± 45.4^c^	5.4 ± 1.5^c^	5935.9 ± 669.2^b^	8665.4 ± 572.5^a^
*Tcp11*	23.9 ± 1.6^c^	26.9 ± 1.3^c^	1256.9 ± 243.3^b^	4906.3 ± 307.0^a^
*Efhc1*	46.7 ± 10.8^c^	40.3 ± 4.8^c^	829.2 ± 175.9^b^	1299.2 ± 58.4^a^
*Tcfl5*	68.8 ± 8.0^b^	139.0 ± 14.1^b^	845.1 ± 201.5^a^	1272.3 ± 50.8^a^
*Zpbp*	8.8 ± 2.3^c^	7.5 ± 3.1^c^	265.0 ± 91.9^b^	1004.4 ± 33.0^a^
*Actl7a*	29.0 ± 6.1^b^	32.3 ± 6.5^b^	40.6 ± 18.6^b^	1705.5 ± 183.3^a^
*Actl7b*	18.0 ± 4.8^c^	12.0 ± 0.8^c^	427.2 ± 152.6^b^	1892.4 ± 96.4^a^
*Spata6*	96.7 ± 10.8^b^	98.0 ± 8.4^b^	525.0 ± 103.2^a^	687.2 ± 28.6^a^
*Ybx2*	10.7 ± 1.8^c^	19.5 ± 4.8^c^	1957.4 ± 601.1^b^	4059.9 ± 158.9^a^
*Zmynd10*	6.1 ± 1.5^b^	5.3 ± 0.9^b^	1574.3 ± 553.7^a^	989.3 ± 70.0^a^
*Stag3*	6.6 ± 0.9^c^	177.8 ± 30.1^b^	611.0 ± 93.2^a^	560.8 ± 11.0^a^
*Odf1*	62.7 ± 9.4^b^	51.8 ± 6.3^b^	72.0 ± 9.6^b^	5039.2 ± 517.1^a^
*Gapdhs*	37.4 ± 4.1^b^	33.9 ± 5.2^b^	38.0 ± 3.8^b^	4990.9 ± 434.5^a^
**Non-testis-enriched genes**				
*Col1a2*	3456.8 ± 240.3^a^	2132.2 ± 104.3^b^	604.7 ± 72.6^c^	280.0 ± 29.2^c^
*Zbtb16*	98.2 ± 19.2^b^	254.2 ± 18.0^a^	61.7 ± 16.1^bc^	32.8 ± 2.9^c^

Means ± SEM are shown. Different superscript letters indicate significant differences between developmental time points.

In addition, our further data analysis showed that, compared to fertile normal males, expression of these testis-enriched genes was significantly decreased in teratozoospermic patients with abnormal sperm morphology according to GDS2697 (S1 Table). It appears that those testis-enriched genes are involved in normal testis development without morphological defects and may serve as a biomarker for teratozoospermic condition. Moreover, according to GDS3906, polyubiquitin knockout resulted in a decreased expression pattern of testis-enriched genes at 28 dpp compared to wild-type (S1 Table).

### Testis-enriched genes are associated with various biological pathways in sperm

**Fig 4:**
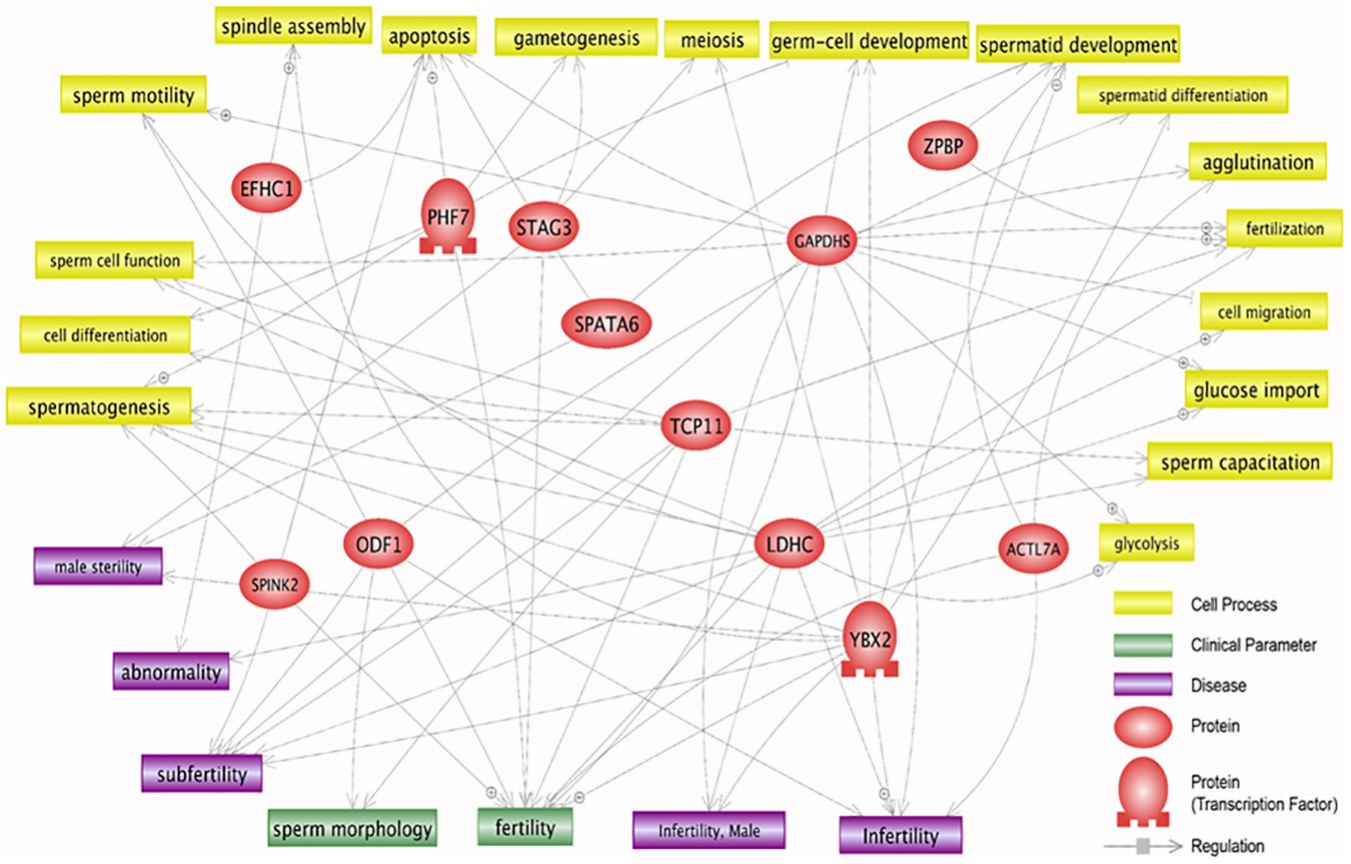
Signaling pathways associated with testis-enriched proteins. The pathway analysis was conducted using Pathway Studio (v 11.2.5.9) following a database search based on PubMed/Medline hyperlink. Pathway inhibition is indicated with flat-headed lines, and activation with arrow-headed lines.

Signaling pathway analysis was conducted to identify corresponding pathways related to sperm-related biological functions and disease conditions. Schematic illustration was drawn to identify cellular and metabolic processes regulated by testis-enriched genes and showed that at least 12 out of 15 spermatocyte- or spermatozoa-enriched proteins were putatively associated with various sperm-related cell processes, clinical parameters, and disease conditions (Fig 4).

## Discussion

In this study, testis-enriched genes in human and mouse were arranged based on microarray based-GEO database, and 15 genes that have not been published regarding their localization in human (Table 2) were selected to analyze their protein expression in testis using immunohistochemical data from the Human Protein Atlas portal.

### Several proteins expressed in pachytene spermatocytes and round spermatids of human testis were analyzed

Proteins localized in pachytene spermatocytes (PC) and round spermatids (RS) were shown in Fig 2A and 2C. PHF7 is a male-specific transcription factor for germ cell development and sexual identity [16, 17]. SPINK2 is a Kazal-type serine protease inhibitor or an acrosin-trypsin inhibitor that is synthesized in the testis [18–20]. LDHC is an enzyme related to aerobic glycolysis in spermatozoa for energy production, and it regulates the sperm motility and capacitation [21]. Based on its localization, we postulated that LDHC is associated, not only with ATP generation in mature spermatozoa, but also with development of germ cells. TCP11 is a receptor of a fertilization promoting peptide that regulates sperm capacitation in the mouse [22]. EFHC1 has been found in mouse sperm flagella and is present in motile cilia, but not in immotile cilia [23]. In this study, EFHC1 was expressed in cytoplasmic regions of testicular cells. It suggests that EFHC1 may be associated with germ cell development and sperm motility. TCFL5 has been found in the testis during spermiogenesis, and it is associated with spermatogenesis and the formation of sperm flagellum in the mouse [24]. YBX2, also known as contrin, is a germ cell specific protein and required for the formation of functional spermatozoa and has been implicated as a potential cause of azoospermia [25, 26]. ZMYND10 has been found in motile cilia of *Drosophila*, and it is associated with male fertility [27]. STAG3 is the meiosis-specific cohesion subunit and is associated with meiotic division of gametes [28].

### Some proteins expressed in the acrosome or cytoplasmic region of spermatids of human testis were presented

The acrosome reaction is required for zona pellucida penetration and fertilization with oocytes [29], and four proteins, ZPBP [30], ACTL7A (T-ACTIN2), ACTL7B (T-ACTIN1), and SPATA6, were localized in the acrosome or cytoplasmic region of spermatids (Fig 2B), implicating their roles in fertility. Other proteins that may also be involved in spermatogenesis during the spermatid phase are shown in Fig 2D. ODF1 is one of the heat shock proteins that play an important role as molecular chaperones in spermatozoa, and it is located in the sperm tails and supports the flagella motility [31, 32]. GAPDHS is a testis-specific glycolytic enzyme and generally known to be present in the principal piece of spermatozoa, and it is associated with ATP production and flagella motility and capacitation [33, 34].

### Expression of testis-enriched genes increased during normal testis development

Those selected testis-enriched proteins were mostly expressed in cells in the late spermatogenesis stages. The stage-specific mRNA expression of these genes showed similar patterns as shown in the GDS2390 dataset (Table 3). Their expression was further analyzed during the testis development. During postnatal testicular growth, the proportion of germ cell types in seminiferous tubules changes. Before 10 dpp, testes of the mouse (Mus musculus) contain mostly spermatogonia. Between 21 and 24 dpp (weaning ages), spermatocytes become the most abundant cell type, round spermatids develops as the most advanced germ cells, and in adults, spermatids are a predominant cell type stage [35, 36]. Based on the changes in types of spermatogenic cells, qPCR was performed at the stage of spermatogonia (10 dpp), spermatocytes and round spermatids (21 dpp), and spermatids (91 dpp). Our qPCR data showed that expression of these testis-enriched genes was significantly increased around weaning ages when spermatocytes and round spermatids are present in testis (Fig 3). Some of them were further increased at 91 dpp when the mouse is sexually matured and spermatids are the predominant form of spermatogenic cells. These expression patterns were consistent with gene expression profiles in GDS605, GDS606, and GDS607 (Table 4). These results suggest that testis-enriched genes may be involved in advanced stages of spermatogenesis when spermatocytes and spermatids are dominant types of spermatogenic cells.

### Testis-enriched genes tend to be repressed in diseases associated with male infertility

Expression of all of these testis-enriched genes was decreased in teratozoospermic patients compared to normal individuals (S1 Table). Spermatogenic cells are susceptible to impairment which causes spermatogenic cells to become arrested at a certain developmental stage. For example, spermatogenic arrest at spermatogonia leads to total germ cell depletion and Sertoli cell only (SCO) syndrome with a lack of germ cells, arrest at spermatocytes gives rise to azoospermia (no spermatozoa) and oligozoospermia (a reduced number of spermatozoa), and arrest at spermatids results in teratozoospermia (an abnormal shape of spermatozoa) [37]. Thus, decreased expression of genes enriched in spermatids in this study could be used as biomarkers for spermatid arrest, teratozoospermia, and subsequent infertility. In addition, polyubiquitin knockout mice showed a decreased expression of these testis-enriched genes compared to wild-types (S1 Table). The ubiquitin-proteasomal pathway (UPP) has been regarded to be a critical process for the successful maturation of spermatids into spermatozoa by tagging and degrading proteins related to morphological defects [38]. In addition, post-testicular presence of ubiquitin plays a role in disposal of defective mature spermatozoa [39, 40]. It has been reported that total knockout of the polyubiquitin gene in mice resulted in a developmental arrest of spermatogenesis followed by infertility [41]. Therefore, the decreased expression of testis-enriched genes in polyubiquitin knockout models can be used as an indicator of failure in sperm maturation. A recent study has shown that knockout mice lacking several testis-enriched genes were fertile [42]; however, the relationship between these genes and normal testis development remains to be explored. Genes presented in the current study that are related to testis development may provide appropriate targets for future knockout studies.

### Various signaling pathways in sperm are linked to testis-enriched genes

Pathway analysis, in this study, provided comprehensive insight into the underlying biological functions and diseases involved in spermatocyte- or spermatozoa-enriched expression. As such, most of spermatocyte- or spermatozoa-enriched proteins being analyzed were implicated in a variety of sperm functions, including motility and capacitation, and multiple disease conditions such as infertility. On the other hand, three proteins (ZMYND10, ACTL7B, and TCFL5) out of those 15 spermatocyte- or spermatozoa-enriched proteins were not implicated in the biological conditions possibly due to incomplete functional annotations (Fig 4).

In conclusion, testis-enriched genes were found based on GEO profiles, and among them, protein localization of 15 genes was identified using the Human Protein Atlas. Mostly, these testis-enriched proteins were expressed in spermatocytes and/or round spermatids, and their expression significantly increased during testis development. In testicular disease conditions, expressions of these genes were significantly decreased suggesting their relation to normal spermatogenesis and testis development. Moreover, in our pathway analysis, most of these proteins exhibited multiple biological implications related to sperm function. Future studies should ascertain the potential involvement of these testis-enriched genes in male infertility.

## Supporting information

S1 Table Microarray analysis of testicular transcriptome. Samples were derived from normal and teratozoospermic individuals aged 21–57 (GDS2697) and from wild-type and polyubiquitin knockout mice at 28 dpp (GDS3906).
(DOCX)

S2 Table Primer sequences for RT-PCR and real-time PCR. (DOCX)
