# Supplementary material for: Comparative expression profiling of testis-enriched genes regulated during the development of spermatogonial cells
Source: PLoS One. 2017 Apr 17;12(4):e0175787. doi: 10.1371/journal.pone.0175787 (PMC5393594; doi:10.1371/journal.pone.0175787)
Supplement: S1 Table — Samples were derived from normal and teratozoospermic individuals aged 21–57 (GDS2697) and from wild-type and polyubiquitin knockout mice at 28 dpp (GDS3906). (DOCX) [file pone.0175787.s001.docx]

**S1 Table. Microarray analysis of testicular transcriptome.** Samples were derived from normal and teratozoospermic individuals aged 21-57 (GDS2697) and from wild-type and polyubiquitin knockout mice at 28 dpp (GDS3906)

| **Gene** | **Normal men** | **Teratozoospermic men** | **Gene** | **Wild-type mouse** | **Polyubiquitin KO mouse** |
| --- | --- | --- | --- | --- | --- |
| **Age** | **21-57 yrs** | **21-57 yrs** | **Age** | **28 dpp** | **28 dpp** |
| *PHF7* | 1119.1 ± 260.4 ^**^ | 138.5 ± 29.4 | *Phf7* | 4024.0 ± 26.1^***^ | 707.0 ± 25.9 |
| *SPINK2* | 2557.7 ± 360.6 ^***^ | 128.8 ± 45.4 | *Spink2* | 2446.7 ± 149.4^**^ | 83.6 ± 21.2 |
| *LDHC* | 1486.2 ± 297.3 ^***^ | 144.7 ± 63.2 | *Ldhc* | 6291.5 ± 161.8 ^**^ | 1225.0 ± 121.7 |
| *TCP11* | 9041.2 ± 460.4 ^**^ | 3955.5 ± 1100.4 | *Tcp11* | 2366.4 ± 282.2 ^0.07^ | 56.5 ± 8.6 |
| *EFHC1* | 1150.9 ± 186.5 ^***^ | 59.7 ± 17.2 | *Efhc1* | 1847.7 ± 111.2 ^*^ | 172.8 ± 5.3 |
| *TCFL5* | 2247.2 ± 424.3 ^***^ | 283.6 ± 87.2 | *Tcfl5* | 3093.6 ± 1.9 ^**^ | 1089.4 ± 72.9 |
| *ZPBP* | 3146.8 ± 394.3 ^***^ | 77.1 ± 25.4 | *Zpbp* | 2225.8 ± 210.3 ^0.06^ | 95.9 ± 8.3 |
| *ACTL7A* | 890.8 ± 131.2 ^***^ | 115.2 ± 19.0 | *Actl7a* | 877.1 ± 111.2 ^0.08^ | 3.0 ± 1.9 |
| *ACTL7B* | 166.3 ± 30.2 ^**^ | 58.9 ± 19.9 | *Actl7b* | 2550.0 ± 117.3 ^*^ | 69.2 ± 0.4 |
| *SPATA6* | 5991.4 ± 685.4 ^***^ | 736.5 ± 345.7 | *Spata6* | 1434.2 ± 33.0 ^***^ | 124.3 ± 3.8 |
| *YBX2* | 1480.9 ± 215.4 ^***^ | 75.23 ± 22.7 | *Ybx2* | 3079.1 ± 407.4 ^*^ | 506.2 ± 55.5 |
| *ZMYND10* | 179.8 ± 21.2 ^***^ | 37.0 ± 7.4 | *Zmynd10* | 1483.3 ± 129.9 ^0.06^ | 121.8 ± 4.3 |
| *STAG3* | 40.6 ± 5.9 ^***^ | 12.6 ± 2.5 | *Stag3* | 1623.6 ± 4.3 ^**^ | 710.3 ±82.0 |
| *ODF1* | 7344.9 ± 733.7 ^**^ | 2949.5 ± 849.6 | *Odf1* | 723.7 ± 12.9 ^*^ | 3.9 ± 0.2 |
| *GAPDHS* | 1133.2 ± 213.0 ^***^ | 143.1 ± 35.7 | *Gapdhs* | 1159.5 ± 198.7 ^0.11^ | 31.8 ± 8.4 |

Means ± SEM are shown. Different superscript letters indicate different *p* values. * *p* < 0.05, ** *p* < 0.01, *** *p* < 0.001.
