# Supplementary material for: Comparative expression profiling of testis-enriched genes regulated during the development of spermatogonial cells
Source: PLoS One. 2017 Apr 17;12(4):e0175787. doi: 10.1371/journal.pone.0175787 (PMC5393594; doi:10.1371/journal.pone.0175787)
Supplement: S2 Table — (DOCX) [file pone.0175787.s002.docx]

| RT-PCR | | Real-time PCR | |
| --- | --- | --- | --- |
| **Mouse** | | Primer Name | Primer Sequence (5’-3’) |
| Primer Name | Primer Sequence (5’-3’) | mPhf7/Real-F1 | CCAAACAGCAAGAAATGGGAAT |
| m-SMCP/F | AACACCGAGAATCAAGTATGGAAA | mPhf7/Real-R1 | CAGAGGACTCGGGCTTTTCTAA |
| m-SMCP/R | AGGACAGGGACAGGGCTTT | mSpink2/Real-F1 | TGAATGTACCCTGTGCATGAAA |
| mOdf1/Real-F1 | TGTGACTACAAGCTGTACTGCCTT | mSpink2/Real-R1 | CTACCCAGAGAAGTTCTGTGAACAA |
| mOdf1/Real-R1 | CGAGGCAGTCGTACCTGTTTT | m-h-Ldhc/Real-F1 | TCAGATAAGGAACACTGGAAAAAT |
| m-CRISP2/F | AAAAGAGCACCCCATATCAACAA | m-h-Ldhc/Real-R1 | CCCAAGACACAAGGGATACTGA |
| m-CRISP2/R | GGAAGGAAAGAAGCCTTGTCATT | mTcp11/Real-F1 | CAGAACATTGCCAAGAAGGAAA |
| mPhf7/Real-F1 | CCAAACAGCAAGAAATGGGAAT | mTcp11/Real-R1 | CAAACACCTGCTGGTTGTGAT |
| mPhf7/Real-R1 | CAGAGGACTCGGGCTTTTCTAA | mEfhc1/Real-F1 | CGGTCGATGACTCGTTGATTA |
| mGapdhs/Real-F1 | CCACGGACTTTAACGGCAAT | mEfhc1/Real-R1 | GAAAGGCTTGGGTGGTAGATTT |
| mGapdhs/Real-R1 | TGGAAGCCGAAGTCAGGAA | mTcfl5/Real-F1 | AGGAAAGACATGGGGACTCTCTTA |
| m-DDX4/F | TGTGCCTCCCAGCTTCAGTA | mTcfl5/Real-R1 | CTCGAGAGGACTCAGAGCTGAA |
| m-DDX4/R | GCAGTGTTGTAACGTCAGCATTT | mZpbp/Real-F1 | GCTTTCCAGGGTATGGCATAAA |
| mZmynd10/Real-F1 | GCTGCTCCAGATGCCAGAAT | mZpbp/Real-R1 | TGGATTGAATGAGGACACCGTA |
| mZmynd10/Real-R1 | GCTGTGCTCACCAGGGAAA | mActl7a/Real-F1 | TTTCCCTAACCGTCTGCAGAA |
| m-Cyc-F3 | GGTGGAGAGAGCACCAAGACAGA | mActl7a/Real-R1 | AGGTTCAGCCCTCACACAGTAAA |
| m-Cyc-R3 | GCCGGAGTCGACAATGATG | mActl7b/Real-F1 | GAGCGTTTCCAGAGGGAGTT |
|  |  | mActl7b/Real-R1 | GCCTCTGCTCTCCAGCTTTT |
| **Human** |  | m-h-Ybx2/Real-F1 | CCTACCACAGAAGGTGGGGAT |
| h-PRM2/F | GACCCATGGCCAGTCTCACTA | m-h-Ybx2/Real-R1 | CTGGTTCCTTCTCAGCCTGA |
| h-PRM2/R | CTTGGGCAGGTGACTTTCTCTT | mZmynd10/Real-F1 | GCTGCTCCAGATGCCAGAAT |
| h-TNP1/F | GATCTCCTCACAAGGGAGTCAA | mZmynd10/Real-R1 | GCTGTGCTCACCAGGGAAA |
| h-TNP1/R | CAGCCAACATATACATTCCTCATTT | mStag3/Real-F1 | AATGGCAACGTGGAGACAA |
| h-SPATA6/F1 | CCTATGACAGTGACCCCGAGTATA | mStag3/Real-R1 | AAAGTACTTGGCTAGGACCCAAA |
| h-SPATA6/R | TCATGGATGGTCTCAGAAGCTTT | mOdf1/Real-F1 | TGTGACTACAAGCTGTACTGCCTT |
| h-NEK2/F | GAAGTTCCTGTCTCTGGCAAGTAAT | mOdf1/Real-R1 | CGAGGCAGTCGTACCTGTTTT |
| h-NEK2/R | CATGGCTCATGGAACCAAGTATT | mGapdhs/Real-F1 | CCACGGACTTTAACGGCAAT |
| m-h-Ldhc/Real-F1 | TCAGATAAGGAACACTGGAAAAAT | mGapdhs/Real-R1 | TGGAAGCCGAAGTCAGGAA |
| m-h-Ldhc/Real-R1 | CCCAAGACACAAGGGATACTGA | mCOL1A2/Real-F1 | GGGGTGTCCTCCAAGGAAAT |
| m-h-Ybx2/Real-F1 | CCTACCACAGAAGGTGGGGAT | mCOL1A2/Real-R1 | GATTGTCTTGCCCCATTCATT |
| m-h-Ybx2/Real-R1 | CTGGTTCCTTCTCAGCCTGA | mZBTB16/Real-F1 | GAGCACACTCAAGAGCCACAA |
| h-EFHC1/F | CCAGTGGATGACTCCTTGGTTAA | mZBTB16/Real-R1 | CTTGTGGCCCTTCATGTGTTT |
| h-EFHC1/R2 | CATCAGCAGGTCAGTTTGAGAAA | m-Cyc-F3 | GGTGGAGAGAGCACCAAGACAGA |
| h-Cyc-F | CTCCTTTGAGCTGTTTGCAG | m-Cyc-R3 | GCCGGAGTCGACAATGATG |
| h-Cyc-R | CACCACATGCTTGCCATCC |  |  |

**S2 Table. Primer sequences for RT-PCR and real-time PCR**
